# The Application of Internal Suspension Technique in Retroperitoneal Laparoscopic Partial Nephrectomy for Renal Ventral Tumors

**DOI:** 10.1155/2017/1849649

**Published:** 2017-05-29

**Authors:** Wenlong Zhong, Yicong Du, Lei Zhang, Xuesong Li, Cuijian Zhang, Dong Fang, Gengyan Xiong, Zhisong He, Liqun Zhou

**Affiliations:** Department of Urology, Peking University First Hospital, Institute of Urology, Peking University, National Urological Cancer Center, Beijing, China

## Abstract

**Objective:**

To evaluate the feasibility of an internal suspension technique in retroperitoneal laparoscopic partial nephrectomy for the management of renal ventral tumors.

**Methods:**

Between January 2013 and July 2016, a total of 145 patients underwent retroperitoneal laparoscopic partial nephrectomy with or without internal suspension technique. For patients who underwent internal suspension technique, the surgeons preserved the external fat of the renal tumor as a suspension traction measure when separating the kidney. Propensity score matching (PSM) was performed according to age, gender, body mass index, tumor size, tumor location, and RENAL nephrometry score. Patient characteristics and intraoperative and postoperative outcomes were compared between the groups.

**Results:**

After PSM, 32 patients treated with the internal suspension technique were compared with 32 cases treated without such technique. Baseline characteristics were statistically similar for the cohorts. The use of our new technique resulted in shorter warm ischemia time (WIT: 15.0 versus 19.0 minutes, *P* = .002) and tumor resection time (4.0 versus 7.5 minutes, *P* < 0.001). The rate of WIT >25 minutes decreased (6.3% versus 25%, *P* = .04) and the trifecta outcomes were significantly improved (87.5% versus 62.5%, *P* = .02).

**Conclusion:**

Internal suspension technique is a feasible and safe procedure in retroperitoneal laparoscopic partial nephrectomy for renal ventral tumors.

## 1. Introduction

With the prevalence of advanced imaging techniques and the enhancement of people's health consciousness, the detection rate of renal cell carcinoma has significantly increased [1]. Despite enhanced understanding of the biological characteristics of renal tumors, surgical resection remains the standard of treatment. For localized renal tumors, partial nephrectomy (PN), as a nephron-sparing surgery, has shown similar oncologic outcomes and additionally improved preservation of renal function compared to radical nephrectomy (RN) [2]. Accordingly, PN now has become the gold standard treatment for T1 renal tumors with a normal contralateral kidney [3]. In 1993, Winfield et al. [4] reported the laparoscopic partial nephrectomy (LPN) surgery for the first time, proving the feasibility of the procedure using the laparoscopic surgical technique. Since then, numerous reports have demonstrated that LPN offers equivalent oncologic outcomes but minimal invasion and quicker recovery compared to open surgery in appropriately selected patients [5, 6].

Although LPN has increasingly become a preferred approach for nephron-sparing surgery, initial experiences are associated with longer warm ischemia time (WIT) due to the technical difficulty [7]. The most critical point of LPN is to resect the tumor and repair the renal parenchyma during WIT. Thus, surgical refinements for more efficient tumor resection and renal reconstruction are of great value.

Regarding innovation in tumor resection, we present a novel technique of “internal suspension” in retroperitoneal LPN (RLPN) for renal ventral tumors. The technique is an intraoperative skill in which surgeons separate the kidney without resecting the external fat of the renal tumors. The adipose tissue between the tumor and Gerota's fascia is preserved and used for suspension traction to stabilize the tumor and generate enough tension during excision. In this study, we present our initial experience and conduct a 1 : 1 matched pair analysis to demonstrate the feasibility of the technique.

## 2. Patients and Methods

### 2.1. Patients Selection

From January 2013 to July 2016, RLPNs performed by the same experienced surgeon were retrospectively reviewed at Peking University First Hospital, Beijing, China. Inclusion criteria were renal tumors ≤7 cm and follow-up for ≥6 months after surgery. Exclusion criteria included solitary kidney, multiple tumors, or history of kidney operation. A total of 145 consecutive patients were included, of which 36 and 109 cases underwent RLPN with or without our internal suspension technique, respectively. Tumor stage and grade were determined according to the 2010 TNM system and the Fuhrman grading system [8, 9]. Trifecta outcomes were defined as the achievement of negative surgical margins, WIT less than 25 minutes, and no perioperative complications [10, 11].

Data on patient characteristics, including age, gender, body mass index (BMI), tumor laterality, tumor size, preoperative estimated Glomerular Filtration Rate (eGFR), American Society of Anesthesiologists (ASA) score, and RENAL nephrometry score [12] and pathological outcomes, were collected. The outcome measures we needed to evaluate the technique included the overall operative time, tumor resection time, WIT, rates of WIT >25 minutes, estimated blood loss, margin status, surgical complications (within 1 month of surgery), postoperative eGFR, follow-up time, and rates of trifecta accomplishment.

### 2.2. Surgical Technique

**Figure 1:**
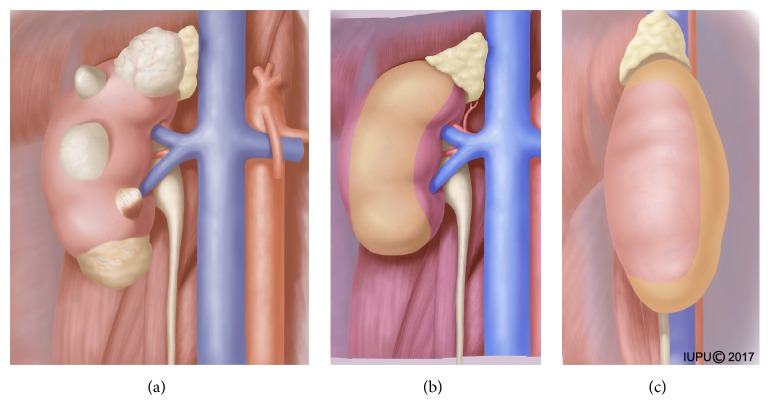
The application range of the new technique. (a) Single renal exophytic tumors located in ventral side with different sizes (<7 cm). ((b) and (c)) The yellow color on the kidney represents the area of the tumor location for the technique.

The application of the novel technique was limited to ventral and exophytic tumors (the right side, for example, Figure 1(a)), with the exception of hilar and anterolateral tumors (Figures 1(b) and 1(c)). After induction of general anesthesia, patients were placed in our modified lateral decubitus position [13]. The distribution of the ports was similar to that previously described by us [14]. The first port was placed through a 2 cm transverse incision located 2 cm below the tip of the twelfth rib and the retroperitoneal space was bluntly created with a handmade balloon dilator. After that, one 12 mm camera port was placed 2 cm above the iliac crest at the mid axillary line, and a 5 mm port was placed at the anterior axillary line 2 cm below the costal arch. In addition, a 5 mm trocar was placed in the anterior axillary line about 6 cm beside the camera port when the assistant port was needed.

**Figure 2:**
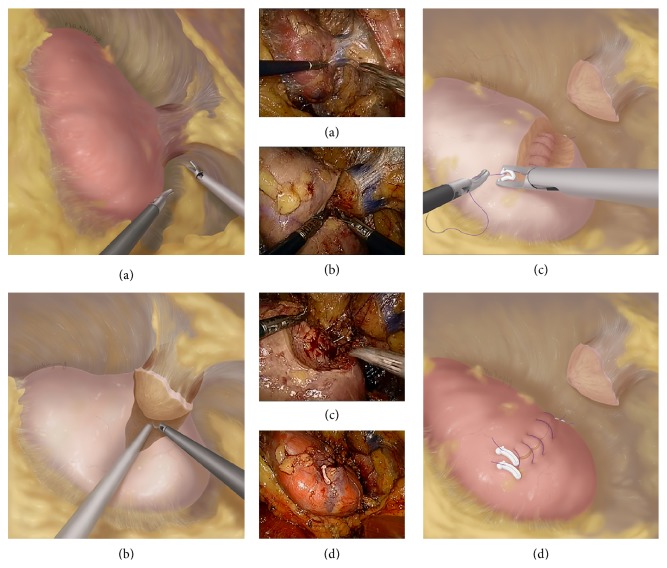
Schematic diagram of the novel technique in RLPN for renal ventral tumors. (a) The perinephric fat along the surface of the kidney was carefully separated without resecting the perinephric fat atop the tumors. (b) The retained perinephric fat exerted traction on the tumor during tumor resection. (c) The closure of blood vessels and collecting system was performed with a unidirectional barbed suture. (d) The second layer suture was performed to close the parenchyma with the same suture.

After that, the retroperitoneal fat was removed and Gerota's fascia was then incised. For the internal suspension traction group, the perinephric fat along the surface of the kidney was carefully separated without resecting the perinephric fat atop the tumors (Figure 2(a)). The perinephric fat was preserved to exert traction on the tumor during resection (Figure 2(b)). For the control group, the perinephric fat was dissected thoroughly without preserving the fat between the tumor and Gerota's fascia.

When the renal artery was circumferentially mobilized, an amount of 12.5 g mannitol was administered intravenously. Once the renal artery was clamped, the tumor was excised using cold scissors with a 5 mm margin of normal renal parenchyma. We closed the blood vessels and collecting system with a unidirectional barbed suture preloaded with a knot and Hem-o-lok clip. After the final tissue bite, a securing clip was applied at the free end of the suture (Figure 2(c)). Then, the second layer suture was continuously performed with a 0-0 barbed suture to close the edge of the parenchyma by the same method (Figure 2(d)). The clamp was removed and the tumor bed was examined for good hemostasis. Finally, the tumor was removed from the perinephric fat and taken out with a specimen bag.

### 2.3. Statistical Analysis

Propensity score matching was performed according to age, gender, tumor size, tumor location, and RENAL score in the groups. Patients undergoing RLPN with the novel procedure were matched 1 : 1 with patients undergoing conventional RLPN. Statistical analysis was performed using SPSS software version 20.0 (SPSS, Chicago, IL, USA). Descriptive data are presented as frequency and percentages. Continuous parametric variables are shown in the form of mean ± standard deviation. Nonparametric variables were expressed as median (interquartile range). Pearson's chi-square test or Fisher's exact test was used to access categorical variables. Mann–Whitney *U* tests were performed for continuous variables. A two-sided *P* < 0.05 was taken to indicate statistically significant difference.

## 3. Results

In the matched cohort, 32 patients who underwent tumor resection with the internal suspension technique were compared with 32 matched cases without the technique. The data of the clinical and pathological characteristics are summarized in Table 1. All of the RLPNs were successfully performed without conversion to open surgery or RN. The matched groups were well balanced in terms of age, gender, BMI, tumor location, tumor size, and RENAL score. No significant differences were observed between the groups for patient tumor laterality, ASA score, preoperative eGFR, and pathological outcomes.

The data of the perioperative, oncologic, and functional outcomes are summarized in Table 2. The use of the internal suspension technique resulted in shorter tumor resection time (4.0 versus 7.5 minutes, *P* < .001) and WIT (15.0 versus 19.0 minutes, *P* = .002). Moreover, the rate of WIT >25 minutes decreased when using our technique (6.3% versus 25%, *P* = .04). The operative time (*P* = .12), estimated blood loss (*P* = .21), positive surgical margins (*P* = .15), follow-up time, and overall surgical complications (*P* = .39) did not significantly differ between the two groups. No major complications (grade ≥ 3) occurred according to the modified Clavien-Dindo classification [15]. Postoperative complications for the internal suspension traction group and control group, respectively, included infection (grade 2, 2 versus 1) and hematuria (grade 1, 0 versus 3). The trifecta outcomes with the novel procedure were significantly improved (87.5% versus 62.5%, *P* = .02). At the last follow-up, the postoperative eGFR was comparable between the two groups.

## 4. Discussion

With the introduction of the laparoscopic technique, LPN has become increasingly widespread for the management of small renal masses. Based on the equivalent oncologic outcomes and improved convalescence, LPN is steadily becoming the accepted option for T1 tumors when technically possible [16]. Both transperitoneal and retroperitoneal approaches have been used for LPN. The surgical approach is chosen primarily based on the tumor location and the surgeon's experience [17]. Most western laparoscopic surgeons prefer the transperitoneal approach for improved working space and clear anatomic landmarks. Conversely, the retroperitoneal approach is more popular among Chinese urologists [18, 19]. As mentioned earlier, retroperitoneal approach offers direct access to the renal hilum and reduces abdominal interference. Furthermore, the approach is more applicable to Asian patients due to less retroperitoneal fat compared with western patients [18]. A recent meta-analysis by Fan et al. [20] found that RLPN had a shorter operating time and a shorter length of hospital stay than transperitoneal LPN. Therefore, we regard the retroperitoneal approach as a preferable choice for LPN.

Either for the transperitoneal approach or for the retroperitoneal approach, the key steps of the LPN are to complete the tumor resection and renal reconstruction with hilar clamping in a time-sensitive manner. Recent studies suggest that every minute of WIT has a deleterious impact on renal function and WIT is the primary surgically modifiable factor in minimizing loss of renal function [21, 22]. Nevertheless, when managing ventral tumors by the retroperitoneal approach, the directional restriction of the laparoscopic operation channel may account for technical challenges with potentially longer WIT. In the past few years, increased attention has been placed on minimizing the WIT during RLPN for the management of ventral tumors [19, 23]. Based on our experience with RLPN in ventral tumor, it is hardly possible to immobilize the tumor and maintain the traction during excision when the perirenal fat is completely separated. In our series, we used a novel technique, namely, the “internal suspension” method, to simplify the resection of renal ventral tumors by RLPN.

The concept of using suspension traction in LPN was described by Chien et al. in 2005 [24]. They presented a renal-suspension traction system to place the tumor in stable optimal view. The method facilitated precise and efficient tumor excision and parenchymal reconstruction, but the placement of suspension traction sutures was complex with the demand for additional procedures. Our technique shares the use of suspension traction in LPN, but, unlike the technique in the previous report, perirenal fat between the tumor and Gerota's fascia was preserved and used as internal suspension traction system. This technique is simple and direct and requires neither assisted devices nor extreme surgical proficiency.

Separating the kidney without resecting the external fat of the renal tumor is the most critical procedure of our technique. With our approach, we could stabilize the tumor in position and maintain the traction during tumor incision without adding a fourth trocar. The method enhanced the ease and speed of tumor resection. Among the 32 patients, it normally took 4 minutes (range: 1–11 mins) to finish resecting the tumor and a WIT of less than 25 minutes was achieved in 30 patients (93.8%). Not surprisingly, when the matched-pair comparison was drawn between the groups, a significant decrease of the tumor resection time was observed in the group with internal suspension. Most importantly, shorter WIT with a decrease rate of WIT >25 minutes was achieved with this technique, which may result in better renal function recovery.

Compared with conventional RLPN, another potential advantage of our novel technique is a better achievement of cancer cure. With the application of our technique, the precision and stability of the tumor excision were improved, which, in turn, may reduce the risk of cutting into the tumor capsule. In our study, all the patients who underwent our new procedure had negative surgical margins on histology. None of the patients showed evidence of local recurrence or metastatic disease at a median follow-up of 21 months. Though there was no significant difference in the rates of positive surgical margins between the two groups, we believed that the lack of significance was probably the result of the small sample size in the current series.

With regard to the complications in our series, no case treated with the novel technique was transferred to open surgery or RN. Though the slight reduction of overall complication rate was nonsignificant, the trifecta outcomes of the new procedure were significantly improved. However, we identified two cases that were converted to conventional RLPN during the internal suspension procedure, which exposed the limitations of our technique. The first patient had a 2.3 cm ventral tumor located closed to the renal hilum. With the application of our technique, the kidney could not be mobilized and rotated. We found that the exposure of the tumor was unsatisfactory and the work space was too narrow for subsequent excision. Another patient with a BMI of more than 30 kg/m^2^ presented a 3.4 cm tumor. We found that the exposure of the tumor without resecting the perirenal fat was challenging and time-consuming for the patient. Accordingly, in our daily practice, the technique is not appropriate for obese patients and patients with ventral hilar tumors.

Among the major limitations of this study are the retrospective nature and the single-center design. The limited sample size and short-term follow-up may also reduce the strength of the study. In addition, due to the preference of the surgeon, we could not compare our technique in LPN to the transperitoneal approach. To further evaluate this technique, larger multi-institutional studies with longer follow-up periods are needed.

## 5. Conclusions

Managing the renal ventral tumors with RLPN is challenging even for skilled surgeons. The internal suspension technique exerts traction on the ventral tumor which would stabilize the tumor and maintain such tension during tumor resection. Our technique is a feasible and safe procedure in RLPN for the management of renal ventral and exophytic tumors. The technique appears to significantly shorten the tumor resection time and WIT, and it enables precise tumor resection with adequate surgical margins. However, larger prospective studies with longer follow-up periods are warranted to confirm the value of the technique.

## Figures and Tables

**Table 1:** Clinical and pathological characteristics of patients with and without natural suspension technique.

	Natural suspension group (*N* = 32)	Control group (*N* = 32)	*P* value
Age (years), mean ± SD	60.7 ± 9.7	62.8 ± 9.2	.23
Gender, *n* (%)			1.00
Male	18 (56.3)	18 (56.3)	
Female	14 (43.7)	14 (43.7)	
Tumor laterality, *n* (%)			.45
Right	15 (46.9)	18 (56.3)	
Left	17 (53.1)	14 (43.7)	
Tumor location (ventral side), *n* (%)	32 (100)	32 (100)	1.00
BMI (kg/m^2^), mean ± SD	22.3 ± 2.6	22.3 ± 2.9	.89
Tumor size (cm), mean ± SD	2.6 ± 0.97	2.6 ± 1.03	.70
ASA score median (IQR)	2.0 (1.0–2.0)	2.0 (1.0–3.0)	.20
RENAL score median (IQR)	6.0 (5.0–6.8)	6.0 (5.0–7.0)	.90
Preoperative eGFR (mL/min/1.73 m^2^), mean ± SD	84.1 ± 11.6	81.9 ± 11.1	.53
Pathological outcomes			
Histology, *n* (%)			.43
Clear cell	29 (90.6)	28 (87.5)	
Papillary	2 (6.3)	4 (12.5)	
Chrome	1 (3.1)	0	
TNM stage, *n* (%)			1.00
T1aN0M0	29 (90.6)	29 (90.6)	
T1bN0M0	3 (9.4)	3 (9.4)	
Fuhrman (clear cell), *n* (%)			.49
G1	6 (20.7)	9 (32.1)	
G2	16 (55.2)	15 (53.6)	
G3	7 (24.1)	4 (14.3)	

ASA: American Society of Anesthesiologists; eGFR: estimated Glomerular Filtration Rate; IQR: interquartile range; SD: standard deviation.

**Table 2:** Perioperative, oncologic, and functional outcomes of patients with and without natural suspension technique.

	Internal suspension group (*N* = 32)	Control group (*N* = 32)	*P* value
Operative time (min), median (IQR)	78.5 (68.3–91.3)	89.0 (78.3–102.3)	.12
WIT (min), median (IQR)	15.0 (12.3–17.8)	19.0 (15.0–25.0)	.002^**∗**^
WIT >25 minutes, *n* (%)	2 (6.3)	8 (25.0)	.04^**∗**^
Tumor resection time (min), median (IQR)	4.0 (3.0–6.8)	7.5 (5.0–11.0)	<.001^**∗**^
EBL (ml), median (IQR)	53.5 (25.3–70.8)	50.0 (20.0–70.0)	.21
Positive surgical margins, *n* (%)	0 (0)	2 (6.3)	.15
Postoperative complications, *n* (%)	2 (6.3)	4 (12.5)	.39
Follow-up time (months), median (IQR)	21.5 (14.5–29.5)	21.0 (13.5–25.0)	.58
Trifecta accomplishment, *n* (%)	28 (87.5)	20 (62.5)	.02^**∗**^
Postoperative eGFR (mL/min/1.73 m^2^), mean ± SD	77.8 ± 10.1	74.5 ± 7.9	.70

^*∗*^Statistically significant. WIT: warm ischemia time; EBL: estimated blood loss; eGFR: estimated Glomerular Filtration Rate; IQR: interquartile range; SD: standard deviation.

## Abbreviations

PN: Partial nephrectomy
RN: Radical nephrectomy
LPN: Laparoscopic partial nephrectomy
WIT: Warm ischemia time
RLPN: Retroperitoneal laparoscopic partial nephrectomy
ASA: American Society of Anesthesiologists
eGFR: Estimated Glomerular Filtration Rate.
